# Association between the C-reactive protein to albumin ratio and adverse clinical prognosis in patients with young stroke

**DOI:** 10.3389/fneur.2022.989769

**Published:** 2022-11-15

**Authors:** Yang Du, Jia Zhang, Ning Li, Jiahuan Guo, Xinmin Liu, Liheng Bian, Xingquan Zhao, Yanfang Liu

**Affiliations:** ^1^Department of Neurology, Beijing Tiantan Hospital, Capital Medical University, Beijing, China; ^2^China National Clinical Research Center for Neurological Diseases, Capital Medical University, Beijing, China; ^3^Research Unit of Artificial Intelligence in Cerebrovascular Disease, Chinese Academy of Medical Sciences, Beijing, China; ^4^Center of Stroke, Beijing Institute for Brain Disorders, Beijing, China

**Keywords:** C-reactive protein to albumin ratio, prognosis, stroke in young adults, young stroke, inflammation

## Abstract

**Background:**

The inflammatory response plays an important role in ischemic stroke, and the incidence of stroke in young adults has increased rapidly in recent years. The C-reactive protein-to-albumin ratio (CAR) is a new index that reflects the overall inflammatory status of patients with major diseases; however, no studies have reported the relationship between CAR and young stroke.

**Methods:**

The participants' baseline characteristics and laboratory examination results, including CAR, were obtained at admission. The modified Rankin Scale (mRS) scores at the 30-day and 90-day follow-ups were obtained from all patients. All the participants included in the study were classified into four groups according to CAR quartiles (Q1–Q4). Logistic regression was used to analyze the relationship between different CAR levels and adverse outcomes (mRS 3–6 and mRS 2–6). We also plotted receiver operating characteristic curves of CAR for adverse clinical outcomes and calculated the area under the curve and cutoff values.

**Results:**

A total of 630 patients with young stroke were enrolled in the study. In the multivariate logistic regression model, at the 30-day follow-up, the Q3 and Q4 (significantly increased CAR) groups showed an elevated risk of mRS score of 2–6 (odds ratio [OR]: 2.94; 95% confidence interval [CI]: 1.40–6.16, *p* < 0.01; OR: 4.01; 95% CI: 1.88–8.91, *p* < 0.01). At the 90-day follow-up, the Q3 and Q4 groups still showed an elevated risk of an mRS score of 2–6 (Q3, OR: 2.76; 95% CI: 1.30–5.86, *p* < 0.01; Q4, OR: 2.63; 95% CI: 1.22–5.65, *p* < 0.01).

**Conclusion:**

A significantly increased CAR was independently associated with an increased risk of adverse outcomes in young patients with stroke.

## Introduction

Stroke is a devastating disease that affects 15 million patients worldwide each year and is characterized by high rates of mortality and residual disability among survivors (1). Ischemic stroke occurring between 18 and 50 years of age (18 ≤ age ≤ 50) is called “young stroke” (2). In recent years, the proportion of so-called youth strokes has increased rapidly, accounting for approximately 15–18% of all ischemic strokes (3). Because these patients carry important social and family responsibilities and often have a life expectancy of several decades, it is critical to improve disease outcomes and understand the factors that influence them. Current studies suggest that the inflammatory response plays an important role in causing stroke and is implicated in the primary and secondary progression of ischemic lesions, as well as in repair, recovery, and overall outcome after a stroke (4, 5). The inflammatory response after ischemia is a complex process that involves the activation and induction of various inflammatory cells and inflammatory factors.

C-reactive protein (CRP) is a trace protein found in the circulation of healthy individuals. However, as a prototypical member of the acute phase proteins, their concentration can be increased by 100 times or more in response to injury, infection, or inflammation (6). In the acute course of the disease, CRP is mainly produced by hepatocytes in response to cytokines such as interleukin-1 (IL-1), interleukin-6 (IL-6), and tumor necrosis factor, reflecting the inflammatory response of the body (7). In addition, albumin (ALB) is the most abundant protein in the serum synthesized by the liver (8). It is the main determinant of plasma osmotic pressure and decreases in cases of malnutrition and inflammation (9). In recent years, the CRP-to-ALB ratio (CAR) has emerged as a novel biomarker for predicting the mortality and prognosis of critically ill patients with coronary artery disease, severe sepsis, or cancer (10–12). Studies have revealed a relationship between CAR and various inflammatory diseases, such as Crohn's disease and rheumatoid arthritis (13, 14).

However, there are no relevant data regarding CAR in studies on young patients with stroke, which is an indicator of systemic inflammation. Thus, we aimed to investigate the relationship between CAR and the prognosis of young patients with stroke in this study.

## Methods

### Patient selection

This was a prospective observational cohort study. The study was conducted in accordance with the guidelines of the World Medical Association Declaration of Helsinki and was approved by the Ethical Committee of Beijing Tiantan Hospital. Written informed consent was obtained from all the patients.

A total of 691 patients with either an ischemic stroke or transient ischemic attack (TIA) were recruited consecutively from Beijing Tiantan Hospital between 2020 and 2022. All patients met the following criteria: (1) age between 18 years and 50 years (18 ≤ age ≤ 50), (2) acute ischemic stroke diagnosed according to the World Health Organization criteria and confirmed by magnetic resonance imaging or brain computed tomography, (3) within 3 days from the onset of symptoms to enrollment, and (4) informed consent provided by the patient or legally authorized representative. The exclusion criteria were as follows: (1) diagnosis of venous sinus thrombosis, intracerebral hemorrhage, or subarachnoid hemorrhage, (2) disease complicated with major comorbidities or late-stage diseases, and (3) onset-to-door time >3 days.

### Clinical variables

Baseline data were collected, including age, sex, body mass index (BMI), medical history (hypertension, diabetes mellitus, lipid metabolism disorders, atrial fibrillation, coronary heart disease, stroke, and TIA), smoking and drinking status, intravenous thrombolysis, and National Institutes of Health Stroke Scale (NIHSS) score at admission.

Hematologic tests were performed on admission by laboratory personnel blinded to the patients' clinical situations, such as routine blood examination, glucose level, low-density lipoprotein cholesterol (LDL-C), CRP, and ALB. The C-reactive protein-to-albumin ratio (CAR) was measured as the ratio of CRP to ALB. We also documented the responsible circulation of the infarct (anterior circulation, posterior circulation, and both anterior and posterior circulation). In addition, trained neurologists used the Trial of Org 10172 in Acute Stroke Treatment (TOAST) classification to evaluate the etiology of each patient.

### Outcome evaluation

Face-to-face interviews were performed when the patient was discharged, and telephone interviews were performed at 30 and 90-day after stroke onset. The researchers were trained to follow-up with the patients without knowing their baseline information and disease characteristics. The neurological function of each patient was evaluated at follow-up and modified Rankin Scale (mRS) scores were obtained. Poor outcomes were defined as mRS scores of 3–6. In addition, the population we studied was relatively young and had important social responsibilities, and we also analyzed the outcome of mRS scores of 2–6 as the unfavorable prognosis. Furthermore, considering that the prognosis of patients with an imaging-proved ischemic stroke or pure TIA differed greatly, we further analyzed the correlation between CAR and the functional prognosis of patients with ischemic stroke.

### Statistical analysis

Statistical analysis was performed using SPSS software (SPSS Inc., Chicago, IL, USA). Participants were divided into Q1, Q2, Q3, and Q4 groups according to the quartile and median CAR levels. Continuous variables were presented as medians with interquartile ranges (IQR) and categorical variables as proportions. Continuous variables were compared using the Kruskal–Wallis test. The Chi-square test was used to compare categorical variables. Multivariable logistic regression was used to analyze the relationship between CAR and clinical outcomes. Factors entered into the multivariate model were those with *P* < 0.1 as determined by univariate analysis. Odds ratios (OR) and 95% confidence intervals (95% CI) were calculated for each group, with the first quartile (Q1) as a reference for the CAR. Furthermore, we plotted receiver operating characteristic (ROC) curves of CAR for adverse clinical outcomes and calculated the area under the curve (AUC) and cutoff values. The results were considered significant at *p* < 0.05 (two-sided).

## Results

### Baseline characteristics

**Figure 1:**
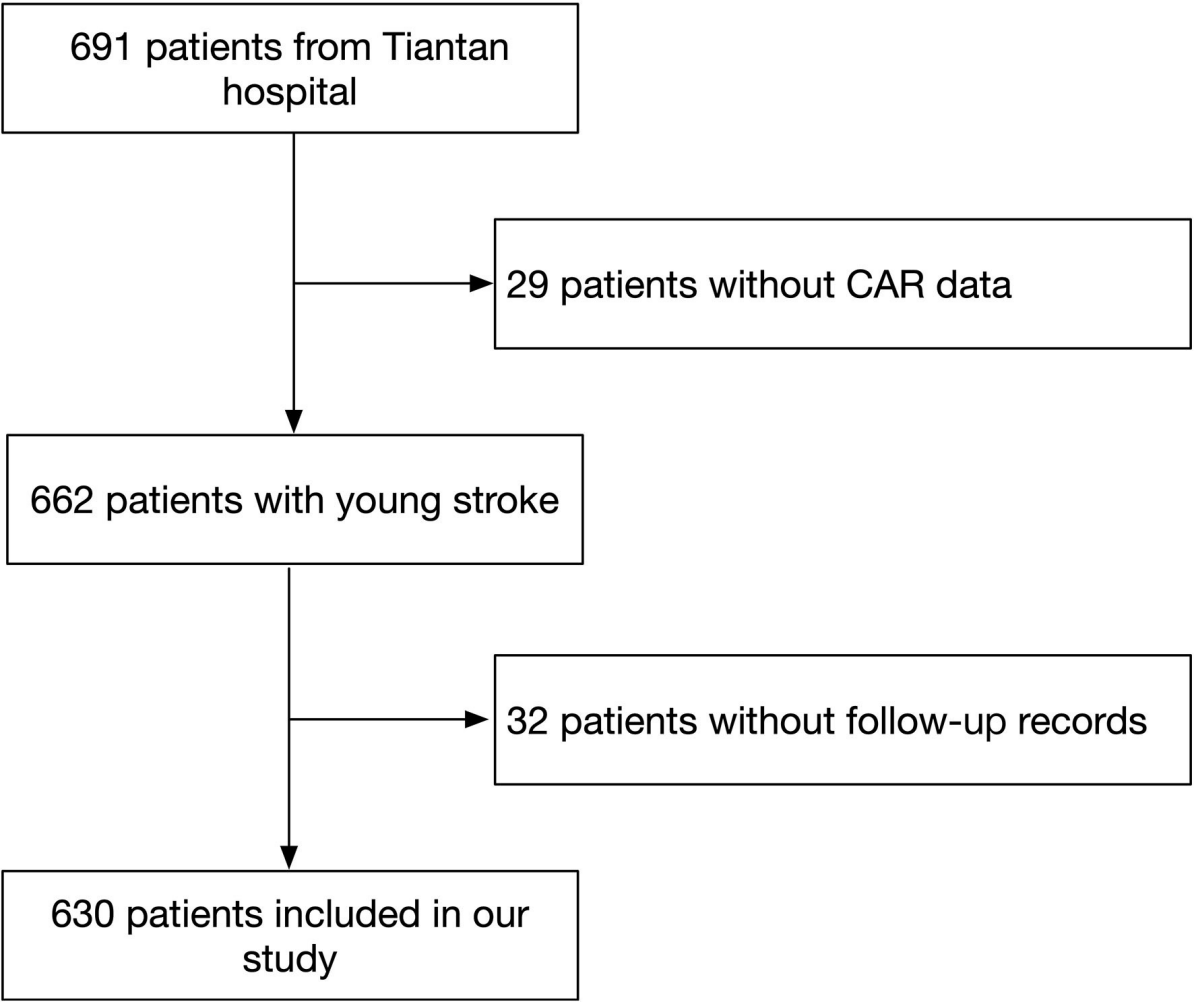
Flow diagram of study patients. CAR indicates C-reactive protein to albumin ratio.

In total, 691 patients were enrolled in this study. We excluded 32 patients without follow-up records and 29 patients without CAR data. Finally, 630 patients were enrolled in this study (Figure 1). The median age was 37 (IQR 33–42) years and 530 (84.1%) patients were men. Participants were classified into four groups by CAR quartiles, and the CAR ranges of the quartile groups were CAR < 0.011, 0.011 ≤ CAR < 0.028, 0.028 ≤ CAR < 0.087, and CAR ≥ 0.087. The baseline characteristics of the patients are shown in Table 1. The median values of age, BMI, NIHSS score, LDL-C, CRP, fasting glucose, leukocyte count, percentage of the male sex, and current smoker increased, whereas ALB decreased with an increase in the CAR level. In addition, hypertension and diabetes were more frequently found in patients with higher CAR. These factors showed significant differences among the four groups (*P* < 0.05).

**Table 1:** Baseline characteristics of the patients stratified by the CAR.

**Variables**	**Total**	**Q1 (CAR < 0.011)**	**Q2 (0.011 ≤ CAR < 0.028)**	**Q3 (0.028 ≤ CAR < 0.087)**	**Q4 (0.087 ≤ CAR)**	***P***
	***N* = 630**	**(*n* = 157)**	**(*n* = 158)**	**(*n* = 158)**	**(*n* = 157)**	
Age, y	37 (33–42)	37 (33–42)	37 (33–40)	39 (33–43)	38 (34–42)	0.040
Male, *n* (%)	530 (84.1)	125 (79.6)	139 (88.0)	135 (85.4)	131 (83.4)	0.221
BMI (kg/m^2^, IQR)	26.28 (24.08–29.33)	24.84 (22.72–26.88)	26.91 (24.67–29.39)	27.03 (24.90–30.16)	27.34 (24.22–30.15)	< 0.001
Smoking, *n* (%)	352 (55.9)	74 (47.1)	94 (59.5)	84 (53.2)	100 (63.7)	0.018
Drinking, *n* (%)	199 (31.6)	43 (27.4)	57 (36.1)	44 (27.8)	55 (35.0)	0.200
Medical history, *n* (%)						
Coronary heart disease	18 (2.9)	3 (1.9)	6 (3.8)	4 (2.5)	5 (3.2)	0.765
Atrial fibrillation	8 (1.3)	2 (1.3)	1 (0.6)	2 (1.3)	3 (1.9)	0.784
Diabetes	156 (24.8)	27 (17.2)	32 (20.3)	46 (29.1)	51 (32.5)	0.004
Hypertension	379 (60.2)	72 (45.9)	95 (60.1)	97 (61.4)	115 (73.2)	< 0.001
Lipid metabolism disorders	542 (86.0)	130 (82.8)	136 (86.1)	141 (89.2)	135 (86.0)	0.437
Intravenous thrombolysis	31 (4.9)	5 (3.2)	12 (7.6)	8 (5.1)	6 (3.8)	0.149
History of stroke or TIA	68 (10.8)	21 (13.4)	14 (8.9)	12 (7.6)	21 (13.4)	0.215
NIHSS at first admission (IQR)	2 (0–5)	1 (0–3.5)	1 (0–4)	2 (0–5)	3 (1–7)	< 0.001
Albumin (g/dL, IQR)	43.30 (41.10–45.60)	43.40 (41.30–45.75)	43.80 (41.80–46.00)	43.60 (41.08–45.65)	42.00 (39.65–45.00)	0.005
CRP (mg/L, IQR)	1.18 (0.49–3.68)	0.25 (0.15–0.38)	0.81 (0.65–1.00)	2.05 (1.48–2.68)	8.88 (5.40–11.72)	< 0.001
LDL-C (mmol/L, IQR)	2.17 (1.61–2.87)	1.84 (1.36–2.43)	2.36 (1.66–3.09)	2.16 (1.68–2.89)	2.33 (1.80–3.09)	0.001
Lymphocytes at first admission (10^9^/L IQR)	1.86 (1.43–2.42)	1.77 (1.33–2.27)	1.91 (1.52–2.50)	1.89 (1.55–2.33)	1.83 (1.33–2.51)	0.554
WBC at first admission (10^9^/L IQR)	7.91 (6.53–9.71)	6.62 (6.61–8.00)	7.57 (6.72–8.83)	8.00 (6.72–9.72)	9.43 (7.89–12.17)	< 0.001
Fasting blood glucose (mmol/L IQR)	5.55 (4.70–7.01)	5.40 (4.57–6.50)	5.50 (4.73–6.55)	5.56 (4.77–7.20)	5.90 (4.90–7.94)	0.062
Circulation of the infarct, n (%)						0.246
Anterior circulation	422 (67.0)	107 (68.2)	105 (66.5)	106 (67.1)	104 (66.2)	
Posterior circulation	197 (31.3)	50 (31.8)	52 (32.9)	47 (29.7)	48 (30.6)	
Both	11 (1.7)	0 (0.0)	1 (0.6)	5 (3.2)	5 (3.2)	
Toast, n (%)						0.192
Large artery atherosclerosis	293 (46.5)	63 (40.1)	63 (40.1)	70 (44.3)	74 (46.8)	
Cardiogenic embolism	44 (7.0)	12 (7.6)	7 (4.4)	10 (6.3)	15 (9.6)	
Small artery occlusion	74 (11.7)	14 (8.9)	20 (12.7)	23 (14.6)	17 (10.8)	
Stroke of other determined cause	132 (21.0)	26 (16.6)	32 (20.3)	38 (24.1)	36 (22.9)	
Stroke of an undetermined cause	87 (13.8)	29 (18.5)	20 (12.7)	22 (13.9)	16 (10.2)	

Values are median (IQR) or number (%).

CAR indicates C-reactive protein to albumin ratio; BMI, body mass index; IQR, interquartile range; TIA, transient ischemic attack; NIHSS, National Institutes of Health Stroke Scale; CRP, C-reactive protein; LDL-C, low-density lipoprotein cholesterol; WBC, white blood cell and TOAST, Trial of Org 10172 in Acute Stroke Treatment.

### CAR and outcomes

Characteristics associated with the patient prognosis are shown in Table 2. A total of 131 (20.8%) patients had poor outcomes (mRS = 3–6) and 217 (34.4%) patients had mRS scores of 2–6 at the 30-day follow-up. The 90-day follow-up results showed that 67 (10.6%) patients had poor outcomes (mRS = 3–6) and 144 (22.9%) patients had mRS scores of 2–6. These prognostic indices showed significant differences among the four groups with different CAR levels (*P* < 0.05).

**Table 2:** Clinical outcomes of the participants stratified by CAR.

**Outcome**	**Total *N* = 630**	**Q1 (CAR < 0.011)**	**Q2 (0.011 ≤ CAR < 0.028)**	**Q3 (0.028 ≤ CAR < 0.087)**	**Q4 (0.087 ≤ CAR)**	***P***
	**(*n* = 157)**	**(*n* = 158)**	**(*n* = 158)**	**(*n* = 157)**		
30-day follow-up						
mRS≥3 (3–6)	131 (20.8)	20 (12.7)	25 (15.8)	37 (23.4)	49 (31.2)	< 0.001
mRS≥2 (2–6)	217 (34.4)	34 (21.7)	43 (27.2)	60 (38)	80 (51)	< 0.001
90-day follow-up						
mRS≥3 (3–6)	67 (10.6)	7 (4.5)	9 (5.7)	19 (12.0)	32 (20.4)	< 0.001
mRS≥2 (2–6)	144 (22.9)	19 (12.1)	29 (18.4)	41 (25.9)	55 (35.0)	< 0.001

Values are number (%).

CAR indicates C-reactive protein to albumin ratio and mRS, modified Rankin Scale.

The risks of adverse clinical outcomes in the CAR quartile groups are presented in Table 3. Compared with the lowest quartile group (Q1) of CAR, the third (Q3) and fourth (Q4) quartile groups had 2.01-fold (95% CI: 1.15–3.80, *p* = 0.02) and 3.11-fold (95% CI: 1.74–5.54, *p* < 0.01) increased risks of an mRS score of 3–6 at the 30-day follow-up, respectively. However, after adjustments, only Q3 remained a significant independent indicator of an mRS score of 3–6 (OR: 3.32; 95% CI: 1.02–4.53, *P* = 0.04). Similarly, compared with the lowest (Q1) CAR quartile group as a reference, the third (Q3) and fourth (Q4) quartile groups had 2.22-fold (95% CI: 1.35–3.64, *p* < 0.01) and 3.76-fold (95% CI: 2.30–6.15, *p* < 0.01) increased risks of an mRS score of 2–6 at the 30-day follow-up. In the multivariate logistic regression model, Q3 (OR: 2.94; 95% CI: 1.40–6.16, *p* < 0.01) and Q4 (OR: 4.01; 95% CI: 1.88–8.91, *p* < 0.01) remained significantly independent indicators of an mRS score of 2–6.

**Table 3:** Risks of adverse clinical outcomes stratified by CAR.

	**Overall**	**Q1 (CAR < 0.011)** ** (*n* = 157)**	**Q2 (0.011 ≤ CAR < 0.028)** ** (*n* = 158)**		**Q3 (0.028 ≤ CAR < 0.087)** ** (*n* = 158)**		**Q4 (0.087 ≤ CAR)** ** (*n* = 157)**		***P* trend**
30-day follow-up	630		OR (95% CI)	p	OR (95% CI)	P	OR (95% CI)	P	
mRS≥3 (3–6)	131 (20.8)								
Crude		Ref.	1.29 (0.68–2.43)	0.44	2.01 (1.15–3.80)	0.02	3.11 (1.74–5.54)	< 0.01	< 0.01
Adjusted^*^		Ref.	1.28 (0.60–2.78)	0.52	2.15 (1.02–4.53)	0.04	1.97 (0.93–4.20)	0.08	0.18
Disabled (2–6)	217 (34.4)								
Crude		Ref.	1.35 (0.81–2.27)	0.25	2.22 (1.35–3.64)	< 0.01	3.76 (2.30–6.15)	< 0.01	< 0.01
Adjusted^†^		Ref.	1.69 (0.81–3.52)	0.16	2.94 (1.40–6.16)	< 0.01	4.01 (1.88–8.91)	< 0.01	< 0.01
90-day follow-up									
mRS≥3 (3–6)	67 (10.6)								
Crude		Ref.	1.29 (0.47–3.57)	0.62	2.93 (1.20–7.18)	0.02	5.49 (2.34–12.86)	< 0.01	< 0.01
Adjusted^‡^		Ref.	1.13 (0.37–3.47)	0.83	2.15 (0.77–6.00)	0.14	2.49 (0.91–6.80)	0.07	0.07
Disabled (2–6)	144 (22.9)								
Crude		Ref.	1.63 (0.87–3.05)	0.13	2.55 (1.40–4.62)	< 0.01	3.92 (2.19–7.00)	< 0.01	< 0.01
Adjusted^§^		Ref.	1.78 (0.82–3.83)	0.15	2.76 (1.30–5.86)	< 0.01	2.63 (1.22–5.65)	0.01	0.10

* Adjusted for age, male, smoking, atrial fibrillation, NIHSS at first admission, WBC at first admission, lymphocytes at first admission, fasting blood glucose, and circulation of the infarct.

† Adjusted for age, male, smoking, diabetes, hypertension, NIHSS at first admission, LDL-C, WBC at first admission, lymphocytes at first admission, fasting blood glucose, and circulation of the infarct.

‡ Adjusted for age, male, atrial fibrillation, lipid metabolism disorders, NIHSS at first admission, WBC at first admission, lymphocytes at first admission, circulation of the infarct, and TOAST.

§ Adjusted for age, male, smoking, atrial fibrillation, NIHSS at first admission, LDL-C, WBC at first admission, lymphocytes at first admission, fasting blood glucose, and circulation of the infarct.

CAR indicates C-reactive protein to albumin ratio; OR, odds ratio; CI, confidence interval; mRS, modified Rankin Scale; NIHSS, National Institutes of Health Stroke Scale; WBC, white blood cell; LDL-C, low-density lipoprotein cholesterol and TOAST, Trial of Org 10172 in Acute Stroke Treatment.

At the 90-day follow-up, compared with the lowest CAR (Q1) quartile group as a reference, the third (Q3, OR: 2.93; 95% CI: 1.20–7.18, *p* = 0.02) and fourth (Q4, OR: 5.49; 95% CI: 2.34–12.86, *p* < 0.01) quartile groups showed elevated risks of an mRS score of 3–6. However, after adjustments, the association between CAR and an mRS score of 3–6 was no longer significant. Regarding an mRS score of 2–6, compared with the lowest CAR (Q1) quartile group as a reference, the third (Q3, OR: 2.55; 95% CI: 1.40–4.62, *p* < 0.01) and fourth (Q4, OR: 3.92; 95% CI: 2.19–7.00, *p* < 0.01) quartile groups showed elevated risks of an mRS score of 2–6. The above associations remained significant after adjustments (Q3, OR: 2.76; 95% CI: 1.30–5.86, *p* < 0.01; Q4, OR: 2.63; 95% CI: 1.22–5.65, *p* < 0.01).

In addition, there were 583 patients with ischemic stroke and 47 patients with TIA in this study. We have further analyzed the correlation between CAR and the functional prognosis of patients with ischemic stroke. The results showed that compared with the lowest CAR (Q1) quartile group as a reference, the third and fourth quartile groups both showed elevated risks of an mRS score of 2–6 at the 30-day and 90-day follow-up after adjustments, respectively (Supplementary Table S1, all *p* < 0.05), which were consistent with the results of the association between CAR and prognosis of patients with ischemic stroke or TIA (Table 3).

**Figure 2:**
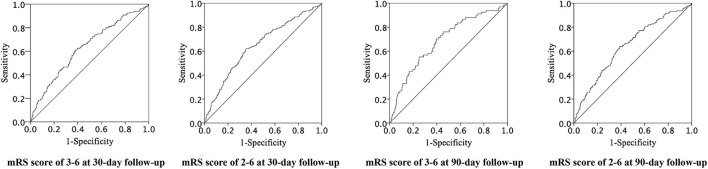
Receiver operating characteristic curves of CAR for adverse clinical prognosis. CAR indicates C-reactive protein to albumin ratio and ROC, receiver operating characteristic.

The ROC curves of CAR for adverse clinical outcomes are shown in Figure 2, and the corresponding AUC and cutoff values are shown in Table 4.

**Table 4:** Predictive values of CAR for adverse clinical outcomes.

**Outcomes**	**AUC (95%CI)**	**CAR cutoff**	**Sensitivity (%)**	**Specificity (%)**
30-day follow-up				
mRS≥3 (3–6)	0.631 (0.577–0.685)	0.036	0.611	0.619
mRS≥2 (2–6)	0.647 (0.601–0.692)	0.034	0.622	0.646
90-day follow-up				
mRS≥3 (3–6)	0.686 (0.618–0.754)	0.036	0.701	0.604
mRS≥2 (2–6)	0.644 (0.593–0.695)	0.034	0.639	0.611

CAR indicates C-reactive protein to albumin ratio; AUC, area under the curve; CI, confidence interval and mRS, modified Rankin Scale.

## Discussion

In this analysis, we found that significantly increased CAR levels correlated with an increased risk of 30 and 90-day mRS scores of 2–6. The correlation between CAR and an mRS score of 3–6 was not significant compared with the former. Our results suggest that given the higher prognostic expectations of young patients with stroke, higher CAR may be an independent risk factor for poor prognosis in these patients.

At present, contrary to the trend of most of the other diseases, the incidence of ischemic stroke in young adults (<50 years old) is increasing and the average age of onset is decreasing (15). Compared with older patients, most young patients with stroke have relatively mild symptoms and better recovery. However, because the patients are young and middle-aged, they bear heavy social and family responsibilities, so they have high expectations for prognosis (16). Therefore, to increase the practical significance of this study and more accurately assess the recovery of patients' symptoms and their ability to return to society and undertake corresponding work, we not only routinely evaluated patients with mRS scores of 3–6 but also considered an mRS score of 2–6 as an important evaluation indicator. The results showed that the effect of significantly elevated CAR (Q3, Q4) on an mRS score of 2–6 remained statistically significant after correcting for relevant confounding factors.

C-reactive protein (CRP) is a trace protein existing in human circulation, with a medium concentration of approximately 1 mg/L and its concentration rises rapidly under the stimulation of trauma, infection, and inflammation (6). Most CRP is produced in the liver. During inflammation, hepatocytes produce CRP under the regulation of inflammatory factors (IL-1, IL-6, and TNF) (7). Current research suggests that CRP has both pro-inflammatory and anti-inflammatory effects (17). C-reactive protein (CRP) activates the classical complement pathway upon ligand binding, which binds to immunoglobulin receptors on immune cells to activate cytokine production and complement-related inflammatory responses, which may exacerbate inflammatory ischemic injury (18, 19). C-reactive protein (CRP) is not only an important marker of acute inflammation but is also involved in the process of chronic inflammation. Because of its complex pro-inflammatory and anti-inflammatory effects, CRP has been shown to have a clear correlation with chronic inflammatory changes in atherosclerosis and is an important risk factor for cardiovascular and cerebrovascular diseases (20). C-reactive protein (CRP) induces various inflammatory changes in endothelial and smooth muscle cells, participates in the uptake of LDL-C by macrophages, and converts them into foam cells (21). Thus, elevated plasma CRP concentrations are found in smokers, those with atherosclerosis, psychological stress, diabetes, or obesity, and older adults (22).

Similarly, low ALB has also been shown to be associated with inflammation (23) and the risk of cardiovascular and cerebrovascular diseases, including acute coronary syndrome and stable coronary heart disease (24, 25). Albumin (ALB) is also produced in the liver. Inflammation and nutritional status not only directly affect the synthesis of ALB but also consume a large amount of ALB at the same time because of the antioxidant and anti-inflammatory properties of ALB. In addition, studies have demonstrated the neuroprotective effect of ALB; that is, sufficient serum ALB is associated with reduced mortality and a better prognosis of ischemic stroke (26, 27). These mechanisms may include the maintenance of osmotic pressure, resistance to blood stagnation and thrombosis, and antioxidant effects (28, 29). The free cysteine-derived redox-reactive thiol (-SH) group (Cys34) can directly scavenge hydroxyl radicals (30), which is the origin of the antioxidant effect of ALB.

C-reactive protein (CAR), which combines CRP and ALB, has become an effective indicator for evaluating the prognosis of various diseases in recent years, including acute coronary syndrome (31), various cancers (hepatocellular carcinoma, gastric cancer, ovarian cancer) (32), and various inflammatory diseases (rheumatoid arthritis, Crohn's disease) (14). Stroke is not only a devastating disease of the central nervous system but also a systemic disease in which the inflammatory response plays an important role because its secondary injury involves multiple body systems. However, as an effective prognostic indicator of inflammation in various diseases, the relationship between CAR and stroke has not been reported. In this study, CAR showed a significant independent correlation with an mRS score of 2–6, which may be related to the intrinsic inflammatory mechanism of stroke.

In the evaluation of prognosis as an mRS score of 3–6, the relationship between CAR and poor prognosis was not significant. The reason may be that there were other factors playing more importance in the poor functional prognosis of patients, and the impact of CAR on the prognosis was relatively weak. Thus, the correlation between CAR and the poor prognosis was covered by those other factors. However, there was a significant correlation between CAR and unfavorable prognosis as an mRS score of 2–6, which was still an important evaluation indicator for young patients compared with older. Therefore, CAR may be an effective predictor of unfavorable functional prognosis in young patients with stroke. In addition, this study was a single-center study, and a multicenter large-scale study will be required to replicate our findings.

## Conclusion

This study revealed that a significantly increased CAR was independently associated with an increased risk of adverse outcomes in young patients with stroke.

## Data availability statement

The raw data supporting the conclusions of this article will be made available by the authors, without undue reservation.

## Ethics statement

The studies involving human participants were reviewed and approved by the Ethical Committee of Beijing Tiantan Hospital. The patients/participants provided their written informed consent to participate in this study. Written informed consent was obtained from the individual(s) for the publication of any potentially identifiable images or data included in this article.

## Author contributions

YD designed the study, interpreted the findings, and wrote the manuscript. YD, JZ, and NL analyzed the data. JG, XL, and LB contributed to data collection and analyses. LB, XZ, and YL provided critical comments/revisions of the manuscript. XZ and YL are responsible for the overall content. All authors contributed to the article and approved the submitted version.

## Funding

This study was supported by the Chinese Academy of Medical Sciences Innovation Fund for Medical Sciences [Grant Number 2019-I2M-5-029]; Beijing Municipal Committee of Science and Technology [Grant Number Z201100005620010]; and Beijing Natural Science Foundation [Grant Number Z200016].

## Conflict of interest

The authors declare that the research was conducted in the absence of any commercial or financial relationships that could be construed as a potential conflict of interest.

## Publisher's note

All claims expressed in this article are solely those of the authors and do not necessarily represent those of their affiliated organizations, or those of the publisher, the editors and the reviewers. Any product that may be evaluated in this article, or claim that may be made by its manufacturer, is not guaranteed or endorsed by the publisher.
