# Supplementary material for: Association between the C-reactive protein to albumin ratio and adverse clinical prognosis in patients with young stroke
Source: Front Neurol. 2022 Nov 15;13:989769. doi: 10.3389/fneur.2022.989769 (PMC9706598; doi:10.3389/fneur.2022.989769)
Supplement: Supplementary file 1 [file Data_Sheet_1.PDF]

***Du Y et al.*, Association between the C-reactive protein to albumin ratio and adverse clinical prognosis in patients with young stroke**

## SUPPLEMENTAL MATERIALS

**Supplemental Table S1：Risks of adverse clinical outcomes in patients with ischemic stroke stratified by CAR**

| | **Overall** | **Q1 (CAR<0.011) (n=146)** | **Q2 (0.011≤CAR<0.028) (n=145)** | | **Q3 (0.028≤CAR<0.091) (n=146)** | | **Q4 (0.091≤ CAR) (n=146)** | | **P trend** |
|---|---|---|---|---|---|---|---|---|---|
| **30-day follow-up** | 583 | | OR (95% CI) | p | OR (95% CI) | P | OR (95% CI) | P | |
| **mRS≥3 (3-6)** | 131(22.5) | | | | | | | | |
| **Crude** | | Ref. | 1.35(0.71-2.55) | 0.36 | 2.22(1.22-4.06) | <0.01 | 2.97(1.66-5.31) | <0.01 | <0.01 |
| **Adjusted**[*] | | Ref. | 1.29(0.60-2.81) | 0.52 | 2.11(0.99-4.45) | 0.05 | 1.90(0.89-4.05) | 0.10 | 0.22 |
| **Disabled (2-6)** | 217(37.2) | | | | | | | | |
| **Crude** | | Ref. | 1.43(0.85-2.42) | 0.18 | 2.41(1.45-4.01) | <0.01 | 3.61(2.19-5.94) | <0.01 | <0.01 |
| **Adjusted**[†] | | Ref. | 1.76(0.84-3.71) | 0.14 | 2.94(1.39-6.21) | <0.01 | 3.92(1.80-8.54) | <0.01 | <0.01 |
| **90-day follow-up** | | | | | | | | | |
| **mRS≥3 (3-6)** | 67(11.4) | | | | | | | | |
| **Crude** | | Ref. | 1.34(0.49-3.71) | 0.57 | 3.07(1.25-7.54) | 0.16 | 5.25(2.24-12.33) | <0.01 | <0.01 |
| **Adjusted**[‡] | | Ref. | 1.11(0.36-3.43) | 0.85 | 2.10(0.75-5.87) | 0.16 | 2.43(0.89-6.65) | 0.09 | 0.08 |
| **Disabled (2-6)** | 144(24.7) | | | | | | | | |
| **Crude** | | Ref. | 1.72(0.91-3.23) | 0.09 | 2.71(1.48-4.96) | <0.01 | 3.75(2.09-6.73) | <0.01 | <0.01 |
| **Adjusted**[§] | | Ref. | 1.80(0.83-3.90) | 0.14 | 2.72(1.28-5.78) | <0.01 | 2.54(1.18-5.47) | 0.02 | 0.13 |

*: Adjusted for age, male, smoking, atrial fibrillation, NIHSS at first admission, WBC at first admission, lymphocytes at first admission, fasting blood glucose and circulation of the infarct.

†: Adjusted for age, male, smoking, diabetes, hypertension, NIHSS at first admission, LDL-C, WBC at first admission, lymphocytes at first admission, fasting blood glucose and circulation of the infarct.

‡: Adjusted for age, male, atrial fibrillation, lipid metabolism disorders, NIHSS at first admission, WBC at first admission, lymphocytes at first admission, circulation of the infarct and TOAST.

§: Adjusted for age, male, smoking, atrial fibrillation, NIHSS at first admission, LDL-C, WBC at first admission, lymphocytes at first admission, fasting blood glucose and circulation of the infarct.

Abbreviations: CAR indicates C-reactive protein to albumin ratio; OR, odds ratio; CI, confidence interval; mRS, modified Rankin Scale; NIHSS, National Institutes of Health Stroke Scale; WBC, white blood cell; LDL-C, low-density lipoprotein cholesterol and TOAST, Trial of Org 10172 in Acute Stroke Treatment.
